# “It’s so much easier for them to just come to us”: a qualitative study examining the implementation of mobile methadone treatment serving a residential SUD treatment program

**DOI:** 10.1186/s13722-026-00692-0

**Published:** 2026-07-01

**Authors:** David Frank, Samantha J. Harris, Minna Song, Megan Miller, Kristianny Ruelas-Vargas, Allison O’Rourke, Ashly E. Jordan, Brendan Saloner, Noa Krawczyk

**Affiliations:** 1 Department of Social and Behavioral Sciences, School of Global Public Health, New York University, New York, NY 10003 USA; 2 Center for Drug Use and HIV/HCV Research, School of Global Public Health, New York University, New York, NY 10003 USA; 3 Department of Health Policy and Management, Bloomberg School of Public Health, Baltimore, MD 21205 USA; 4 Department of Health Services, Policy and Practice, Brown University School of Public Health, Providence, RI 02903 USA; 5 Department of Population Health, NYU Grossman School of Medicine, New York, NY 10016 USA; 6 New York State Office of Addiction Services and Supports, 501 7th Avenue, New York, NY 10018 USA

**Keywords:** Mobile medcation units (MMU), Methadone maintenance, Mobile methadone, Opioid treatment programs (OTPs), Feasibility studies, Residential treatment programs, Retention

## Abstract

**Background:**

Methadone is a highly effective treatment for opioid use disorder (OUD). Yet its impact is constrained by low rates of treatment initiation and retention, driven in part by geographic inequalities in the availability of methadone-providing opioid treatment programs (OTPs) and restrictions on the types of clinical settings where methadone can be dispensed. In response, in July 2021, the Drug Enforcement Administration released a new rule allowing OTPs to dispense medications for OUD—including methadone—through mobile medication units (MMU) without the need for additional treatment waivers.

**Methods:**

We conducted interviews with 11 participants living in a residential substance use treatment facility in NYC and receiving methadone treatment (MT) from an MMU. Interview data were coded using Dedoose software based on a combination of inductive and deductive coding strategies, and guided by a thematic approach to explore patient’s treatment experiences and perceptions.

**Results:**

Participants described MMU as substantially reducing the logistical burden of treatment while also allowing patients to avoid problems associated with brick-and-mortar OTPs. Some raised minor complaints (i.e., additional waiting time on medication delivery days), yet participants framed these concerns within the context of their overall preference for MMU. Participants also expressed uncertainty about how methadone treatment would continue after leaving residential care, highlighting potential challenges in transitioning from mobile services to traditional clinic settings.

**Discussion:**

Our findings provide qualitative evidence from patients’ perspectives on how mobile methadone delivery can potentially reshape the logistical demands, treatment environments, and continuity-of-care challenges associated with methadone treatment in residential settings.

## Background

Methadone treatment (MT) has been extensively studied for more than 70 years and is among the most effective approaches for reducing unwanted opioid use [1, 2], overdose [3, 4], and all-cause mortality [5]. There is also strong evidence that MT supports patients’ efforts to maintain stable employment, attend school, and strengthen family and social relationships [1, 6, 7]. However, consistently low rates of treatment initiation and retention–due in part to geographic disparities in access as well as restrictions on the types of facilities that can dispense MT [8–10]–limit MT’s potential impact [11, 12]. Moreover, the logistical demands associated with attending opioid treatment programs—including transportation, scheduling constraints, and daily attendance requirements—can create a substantial “treatment burden” for patients [8, 9, 13]. Research across health care settings suggests that such burdens can significantly shape patients’ ability to initiate and sustain long-term treatment engagement [13, 14].

One strategy to improve access has been the recent implementation of mobile medication units (MMU)–motor vehicles with medical equipment that travel to different locations to provide health care services [15–17]. Until recently, federal regulations restricted use of MMUs and only allowed methadone dispensing at brick-and-mortar opioid treatment programs (OTPs) that operate in fixed locations [16, 18]. However, in July 2021, the Drug Enforcement Administration (DEA) released a new rule that allows OTPs to dispense methadone through MMUs by extending an OTP’s DEA license to cover a mobile unit without the need for additional treatment waivers [17, 19]. Although MMUs are each linked to a fixed-site OTP, and travel between that location and another site in the community, they can provide all of the same services as a fixed-site OTPs [20]. Following the DEA regulation change, New York State (NYS) began a state-wide initiative to support OTPs to implement MMUs in hopes to expand equitable access to MT [21].

While data on mobile methadone units (MMUs) in the U.S. remain limited, programs that operated in the 1990s indicate MMUs improved retention in care (relative to patients in fixed site programs) [22] and facilitated increased access for some underserved populations [17, 23]. For example, the New Jersey Medication Assisted Treatment Initiative (NJ-MATI) found that its mobile methadone program reached higher proportions of Black patients, patients experiencing homelessness, uninsured patients, and individuals not recently engaged in treatment compared with the program’s brick-and-mortar location [24]. A similar program that provided mobile buprenorphine treatment also found that patients served by the mobile unit were more likely to belong to historically marginalized racial and ethnic groups and to be experiencing homelessness [25]. Mobile methadone programs have existed outside of the U.S., in countries such as Canada, Portugal, Australia, and others since at least the 1980s [26]. Amsterdam began *“methadone by bus”* programs, with mobile buses dispensing methadone in different neighborhoods in 1980 [27–30]. Because mobile units can be deployed to locations where people who use opioids commonly reside—such as residential treatment programs, nursing homes, homeless shelters, encampments, and correctional facilities—they offer an effective mechanism for delivering methadone treatment to individuals who may have been unable to access care under prior U.S. regulatory frameworks. For example, MMUs may be a particularly effective means of reaching medically institutionalized populations, who are necessarily limited in their ability to travel to OTPs.

For this study, we used interview data to examine the experiences and perceptions of people receiving mobile methadone in a residential substance use disorder (SUD) treatment facility in NYS. We aim to advance understanding of how patients conceptualize the benefits and potential limitations of mobile methadone by examining patient experiences under the post-2021 DEA regulatory context in the United States, and to describe their views on how best to refine and improve upon current efforts.

## Methods

### MMU participant recruitment and interviews

We conducted phone interviews with 11 people who used a MMU in one residential SUD treatment facility in NYC between November 1, 2024 to March 30, 2025. Participants were recruited using a purposive sampling strategy whereby investigators provided flyers with study contact information (phone number) to MMU providers; flyers were then placed in locations accessible to patients. The SUD facility served an urban population. The MMU, began serving patients on November 1, 2024, and served 167 patients during our recruitment period (Nov 1, 2024 and March 30, 2025). The MMU van parked outside of the facility and residents could take their medications on site for storage and management by residential treatment staff. Patients who called the study team were screened for eligibility–participants had to be at least 18 years old, speak English, and have been treated for an OUD in the last year or currently using opioids such as heroin, fentanyl, oxycodone, or prescription opioid pills at least once per week.

A semi-structured interview guide was developed collaboratively based on study aims and relevant literature (i.e. MT; mobile HEALTHcare). Co-investigator Frank, who has over 20 years of experience as a MT patient, played a key role in ensuring that study questions reflected patient perspectives [31, 32]. Cognitive interviews, which involved conducting preliminary interviews to assess and refine interview questions [33] were then conducted with five people who had current or previous experience with methadone treatment, and results were used to further refine interview questions. Domains included: current and previous experience with MMU and MT; perceptions of MT; perceptions of mobile MT; barriers and facilitators to treatment; and plans for treatment after leaving residential care.

Sample size was determined using saturation as a guide [34, 35]. As the same themes began to appear in each interview, we ended the data collection phase of the study. Interviews lasted 30–45 min and were recorded and later transcribed. Participants were paid $60 to compensate them for their time, and this study was approved by Johns Hopkins Institutional Review Board. All participants are referred to by pseudonyms.

### Data analysis

Interviews were transcribed and then coded using Dedoose software [36]. Our analytic approach was informed by reflexive thematic analysis [37–39], situated within a constructivist epistemology [40]. We approached participants’ accounts not as direct representations of objective reality, but as meaning-making processes shaped by institutional contexts, prior treatment experiences, and broader structural conditions governing methadone access. Themes were developed iteratively through a combination of inductive coding grounded in participants’ narratives and deductive coding informed by existing literature on methadone treatment, access, and institutional care environments. Throughout analysis, investigators engaged in reflexive discussion to examine how our disciplinary backgrounds and lived experiences shaped interpretation.

Our analysis was also informed by a patient-centered care framework and scholarship [41–43] examining how regulatory and institutional structures shape methadone treatment experiences [44–46]. We interpreted participant narratives in relation to broader structural conditions—including transportation requirements, clinic culture, regulatory constraints, and continuity-of-care barriers—that influence treatment initiation and retention [9, 11, 44, 47–49].

Intercoder agreement was assessed using a subset of 4 interviews. Each interview was coded independently by two investigators, and then later examined by a third investigator to assess alignment. Both coders were found to have coded the same sections with similar codes on each interview; any discrepancies were minor and were resolved by discussion [50].

### MMU service provision data

Deidentified data on MMU service provision were provided by the NYS Office of Addiction and Substance Abuse Services (NYS OASAS). From these data, we calculated by unique client, number of MMU services, days between MMU services, and length of time received services from MMU (first to last service date) while in Residential Treatment.

## Results

### MMU service provision to residential treatment patients

As of June 30, 2025, the MMU had provided methadone to 239 residential treatment patients, who received medication on average every 7 days with a mean care episode of 87 days (Table 1).

**Table 1:** MMU usage characteristics (through June 30,2025)

	Residential partnership
Total unique clients	239
Number of visits to MMU	
Mean	12.0
Median	9
IQR	14 (4 to 18)
Length of care episode (first to last recorded MMU visit, in days)	
Mean	87.2
Median	70
IQR	105 (28 to 133)
Time between visits to MMU (days)	
Mean	7.5
Median	7
Mode	7
IQR	0 (7 to 7)

The following five themes emerged from the interviews and illustrate how mobile methadone reshaped patients’ experiences of treatment access, clinical interaction, and expectations for continuity of care: (1) Reducing the logistical burden of methadone treatment; (2) Avoiding drug-related triggers and clinic risk environments; (3) More personal and predictable interactions with providers; (4) Operational challenges; and (5) Anticipating the return to restrictive treatment systems. These are described below.

### Theme 1: Reducing the logistical burden of methadone treatment

Participants consistently described methadone treatment as shaped by significant logistical demands, particularly the time, transportation, and coordination required to travel to a brick-and-mortar OTP. Before the MMU was implemented, residents receiving methadone were transported by bus to a clinic located in another part of the city, sometimes on a daily basis. Participants contrasted this experience with the MMU model, which they described as a substantial improvement that reduced many of the practical burdens associated with obtaining medication. As Jerry explained:

When I first started you would actually have to go to the actual [brick-and-mortar] clinic to get [your methadone]. We would go to the clinic once a week and pick up a bottle from the clinic and then take them back to the [residential] program.

“Jerry” (40-year-old male)

All participants reported problems with this model and a strong preference for using MMU. Many described how the process of transporting patients, from the treatment facility to an OTP, was complicated, time consuming, and introduced the potential for several problems, including patients becoming lost or separated from the group.

We would have to go there from the program to the brick-and-mortar clinic with about 15 or 20 patients for us to see the nurse, and get dosed, and whatever else we had to do. So it turns into an all-day trip. People are disappearing and -- It’s so much easier for them to just come to us. We go outside for five minutes. We dose in the van. Then we get our bottles for the week or the two weeks, whatever the case is.

“Christopher” (39 year-old male)

The van is much better. It’s much easier and convenient. We don’t have to get up early in the morning every day to get ready to go. We got a lot of people going at the same time. So [when we had to take a bus to OTP] everybody’s got to wait for everybody. Now we just have the van. So we just go right out front and just get it and come right back in and it’s over.

“Keith” (61-year-old male)

Across interviews, participants framed these trips as time-consuming and unpredictable, often requiring coordination among large groups of residents. In contrast, the MMU allowed patients to obtain their medication quickly and return immediately to the facility, which participants described as reducing disruption to their daily routines. In some cases, the time previously needed to commute to the OTP caused patients to experience withdrawal symptoms. For example, Monte stated:

We had to go to the clinic to get dosed pretty much every day. And sometimes they wouldn’t pick us up till like 12:00 in the afternoon. And then, by the time you get there and get dosed–and I’m used to taking my methadone at 7, 8 in the morning–so come 12 o’clock, you’re not feeling too well.

“Monte” (37-year-old male)

Participants also noted how, in contrast to the chaos and uncertainty that came with commuting to an OTP, they were confident in the ability of the MMU program to consistently deliver their medication.

It’s reliable. I know they’re going to be there. It’s consistent.

Henry (29-year-old male)

We know the van’s going to come. We’re not going to miss the van. A million things could happen not making it to the [brick-and-mortar] clinic, whether it be missing the train or missing your Medicaid cab or Medicaid cab not showing up. And you don’t make it in time.

“Christopher” (39-year-old male)

Not surprisingly, participants reported that MMUs consistency–particularly as compared to the previous method–reduced, or eliminated their anxiety about whether they will receive their medication that day. As “Andrew”, a 34-year-old man, stated:

It helps you get through your day, especially rather than going every day to a program and transiting and worrying about the trains and the buses and whatever else you’re going to run into in the streets.

Taken together, these accounts suggest that the MMU reduced the *burden of treatment*, particularly the time and coordination required for group transportation to OTPs as well as the anxiety patients experienced through transporting patients to and from the brick-and-mortar OTP. In this sense, the MMU did not merely improve convenience; it reshaped the temporal structure of treatment by allowing patients to integrate medication access into their daily routines with minimal disruption.

### Theme 2: Avoiding drug-related triggers and clinic risk environments

Participants frequently contrasted the environments surrounding brick-and-mortar OTPs with the quiet and secluded setting where the MMU operated. Several participants described brick-and-mortar OTPs as places where they would regularly encounter people selling drugs, people with whom they had previously used drugs with, and other drug-related triggers that most participants wanted to avoid. In contrast, receiving methadone through the MMU allowed participants to avoid these encounters. As Monte, a 37-year-old male described:

[MMU is] less stressful, less triggers. I don’t have to worry about running into people that are selling drugs or people I used to get drugs from, seeing people do drugs outside of some of the methadone facilities. It’s a lot less triggers. No triggers, actually, and I feel safe when I’m on the van.

Similarly, Keith described how being at brick-and-mortar OTPs often involved unwanted interactions with people wanting to purchase methadone or other drugs:

You don’t want to see the drug dealers and all that. You don’t want to see that stuff. [At the OTP] You see people high right there and all of that. You don’t want people asking for your shit, for your methadone and all of that. You don’t want to sell that. They try you out there. They ask for your methadone, right? They want to talk about your methadone.

“Keith” (61-year-old male)

At the same time, other participants described how some patients would use the trips to obtain drugs and/or not return to the treatment center.

They [patients] would have people meet them up there and they would get stuff. They might be buying drugs to bring back to the program. The more you’re at the clinic, the more detrimental it is to your recovery because people are selling drugs up at the clinic and they’re hanging outside smoking cigarettes, trying to sell benzos or sell their take-homes, so as much as I could stay away from the clinic is the better.

“Christopher” (39-year-old male)

These accounts suggest that the benefits participants associated with the MMU were not limited to convenience or reduced travel time. Participants also viewed the MMU as altering the broader risk environment surrounding treatment by allowing them to receive methadone without entering spaces where drug use, drug markets, or former using partners were present. In this sense, the MMU functioned not only as a delivery mechanism for medication but also as a way of restructuring the social and environmental contexts in which treatment occurred.

### Theme 3: More personal and predictable interactions with providers

Participants also described how receiving methadone through the MMU shaped their interactions with treatment staff. In contrast to their experiences in larger brick-and-mortar OTPs, several participants noted that the smaller scale of the MMU visits made interactions with staff feel more individualized. As participants explained:

I love it [MMU]. I think it’s much better… The lady’s nice. Get your dose right there and then they get the rest of your bottles. You’re good.

“Keith” (61-year-old male)

I know the people that work there now. So, you know they identify with me a little… The process is a little more personal. The staff actually recognizes you and knows who you are.

“Jerry” (40-year-old male)

Participants contrasted these interactions with their prior experiences in larger OTP settings, where high patient volume and clinic routines sometimes made encounters feel more impersonal. By operating in a smaller and more stable environment, the mobile unit appeared to support interactions that participants perceived as more personal and less bureaucratic than those experienced in traditional clinic settings.

### Theme 4: Operational challenges

Participants did report some concerns with the MMU. The most common issue was that patients were often dosed later than normal on days when medication was delivered to the MMU. For example, one participant who preferred getting their methadone at 7:30 am, described having to wait until 11:30 am or 12:30 pm on days when the MMU came. As “Cole” (55-year-old man) explained:

That would improve the services if they could get there on time every day. And I don’t think that’ll happen. That may not be feasible. But if that could be the case, I would like to get there every day on time so I wouldn’t have to wait.

Participants also noted that dose changes required approval from the OTP physician overseeing their methadone treatment, who was not typically present on-site at the MMU. This sometimes made it difficult for patients to make changes to their treatment. As “Henry”, a 29-year old man, explains:

It’d be nice if the doctor -- maybe they were available through telehealth and could approve of a dose change that day so you don’t have to wait a whole other week. I get comfort meds. And [the residential facility] prescribes those. It would be nice if the clinic prescribes [comfort medications] themselves, so I didn’t have to go to two different doctors.

Although remote dose changes are allowed by telehealth, participants were unaware of this option.

Importantly, waiting can be particularly difficult for patients with insufficient doses since they may experience symptoms of withdrawal as Henry’s account demonstrates:

There was a week where I was feeling sick because my dose was [too low]… I felt really sick for a week. But I couldn’t change my dose till the next week. Actually, till two weeks. Because I see them and tell them I want to change it, and then they change it the next week.

In addition to participants’ desire for the van to arrive earlier and the ability to make changes to their treatment more rapidly, some also suggested including harm reduction services such as a syringe exchange program in the MMU:

I mean, they could have a needle exchange as part of it. That would make sense, I think. I don’t think it would be a contradiction. I mean, if the purpose is to combat this opioid epidemic and to stop the spread of hepatitis and HIV. You know what I mean? And also distributing the little test kits that lets you know if you have fentanyl in the drugs you have. That type of thing.

Darren (55-year old man)

Although participants overwhelmingly preferred the MMU model, their comments highlight how relatively small operational issues—such as delayed arrival times or slow dose adjustments—can have disproportionate effects on patient comfort and engagement. Participants suggested several potential improvements such as: establishing earlier, reliable dosing windows; enabling same-day telehealth approvals for dose changes; and, consolidating prescribing under the MMU clinic.

### Theme 5: Anticipating the return to restrictive treatment systems

Although participants overwhelmingly preferred receiving methadone through the MMU while in residential treatment, many simultaneously expressed concern about how their treatment would continue after leaving the program. For example, “Ashley,” a 32-year-old woman, reported wanting to continue treatment through the same OTP organization after discharge, even if this required transitioning from the MMU to the affiliated brick-and-mortar clinic. However, because she had recently had a baby and the clinic did not allow children on-site, she was uncertain whether remaining with that provider would be feasible. She explains:

I would really like to stay at [redacted]. I’ve just seen the mobile unit today. I was there. I got my bottles today. And, like I said, they don’t allow children at the actual [non-MMU] facility… So I have to go to them and talk to the doctor and see how it’s going to work out next week. I’ve made it very clear to my counselor that I would prefer to stay, and she’s trying to push me in other directions [to another OTP]. And I’m like, ‘Listen, if it’s not broken, don’t fix it.’

For many participants, including Ashley, the desire to continue using mobile services was based in part on previous negative experiences with brick-and-mortar OTPs. Participants described problems obtaining needed transportation consistently, OTP fees that were too expensive, and a clinic culture they perceived as top-down and overly strict, and that made attending brick-and-mortar OTPs difficult and unpleasant. When asked if transportation issues had caused them to miss treatment, participants explained:

Oh, absolutely. Usually, if the treatment doesn’t provide a Metro Card or something of that nature, it could be an issue. Yeah, if you have to go there every day… I’m probably going to have to face that issue in the future because I’m getting ready to set sail or leave here.

“Jerry” (40-year-old male)

At one point, I was taking a dose of 190 milligrams [at a brick-and-mortar OTP], right? I missed three days consecutively because of the medical transportation issue. And even though I explained to the doctor at the clinic that it wasn’t my fault, he changed my dose from 190 to just 20 milligrams. And he said there had to be a punishment for missing three days in a row. That’s my life you’re messing with. So the playing God with my dose thing, I do not like that.

“Ashley” (32-year-old female)

As such, for many patients, use of the MMU was a temporary respite from the less accessible brick-and-mortar clinics, and many expressed significant concerns about how their return to a brick-and-mortar clinic would impact their lives including the progress they have in treatment. As participants stated:

I’m just worried about when I start working, will I be able to get my methadone [and make it to work on time] in the morning? That’s what I’m worried about.

“Keith” (61-year-old male)

I have seven months clean with [residential facility]. I’ve given them seven months of clean drug tests, and that counts for something. I can get take-home bottles. I’ve established a rapport there. If I go to a different clinic, I would have to start that whole process all over again. And that’s just a pain in the butt having to go to the clinic every single day.

“Ashly” (32-year-old female)

These narratives illustrate how MMU may create expectations of more flexible, patient-centered care, that may be less available if patients are forced to return to a brick-and-mortar provider. In this way, MMU may create a potential “treatment cliff,” in which patients transition from a flexible, low-burden service model to a more restrictive clinic environment. This discontinuity may undermine retention even among individuals who had previously stabilized on methadone during residential treatment.

## Discussion

This study examines how patients receiving methadone through a MMU within a residential substance use disorder treatment program experienced this model of care. Participants’ accounts highlighted five key dimensions of this experience: the reduction of logistical barriers to treatment, the ability to avoid drug-related environments often encountered near brick-and-mortar OTPs, the development of more familiar interactions with providers, organizational challenges, and concerns about returning to more restrictive treatment systems after leaving residential care.

Participants repeatedly emphasized the logistical burdens associated with traveling to traditional OTPs, including transportation coordination, time spent commuting, and uncertainty around arrival times. These accounts aligned with literature showing that logistical barriers such as transportation requirements and daily attendance obligations are significant predictors of treatment dropout [9, 51]. The MMU model substantially reduced these burdens by allowing patients to receive medication directly at the residential facility. Participants also described the benefits of avoiding brick-and-mortar OTPs, and noted that the MMU facilitated more familiar interactions with treatment staff. Perhaps unsurprisingly, receiving medication in a smaller and more consistent setting appeared to create opportunities for more personalized interactions than those experienced in larger OTP clinics. Although some participants did report minor problems with MMU, such as a desire for the service to arrive earlier on days when their medication was delivered, they all framed such issues as minor and in the context of their overall preference for the MMU model. Taken together, these findings suggest that mobile methadone services reduce what might be conceptualized as the *treatment burden* of methadone maintenance—namely the time, transportation, and social exposure associated with attending brick-and-mortar OTPs.

Importantly, although MMUs and brick-and-mortar OTPs operate under the same regulatory framework, implementation of take-home dosing practices can vary substantially across programs. Some participants in this study reported received weekly or biweekly take-home medication while residing in the residential facility. However, take-home eligibility is often shaped not only by formal regulations, but also by clinic culture, medical director discretion, staffing capacity, organizational philosophy, and other operational considerations. Consequently, the degree to which MMUs function as lower-burden alternatives to traditional OTPs may depend in part on how individual programs operationalize flexibility within existing regulatory structures.

Mobile units could provide an opportunity to expand methadone access to populations in other institutional settings (including both fixed-site (ambulatory) and institutional/long-term stay settings), such as people who are incarcerated, who comprise a sizeable OUD population often lacking access to evidence-based treatment [52, 53]. A recent study on availability of medication for opioid use disorder (MOUD) in US jails found less than half (43.8%) of the 1,028 jails offered any form of MOUD, and only 12.8% made these available to anyone with OUD [54, 55]. Mobile units could also potentially serve patients in skilled nursing facilities, long-term care facilities, and hospice facilities. Such services will likely become more important to people using MT as the population ages [56].

While participants overwhelmingly preferred receiving methadone through the MMU while in residential treatment, many also expressed concern about returning to community OTPs after discharge. Many described prior negative experiences in brick-and-mortar opioid treatment programs (OTPs) and expressed a clear preference for remaining in a mobile methadone unit (MMU), even while acknowledging the limited availability of such services. These accounts align with literature documenting how patients often perceive brick-and-mortar OTPs as top-down, rigid, and at times punitive [31, 57–59]. Thus, patients receiving methadone through MMUs within residential programs may experience a relatively low-burden and supportive model of care, yet upon discharge they often face a return to more restrictive clinic structures. In this context, it is reasonable to anticipate that some patients who receive MMU-based care in residential settings may discontinue methadone treatment when they are required to transition to a markedly different and potentially less flexible model of care upon discharge [31].

Patients receiving medications for opioid use disorder are particularly vulnerable to treatment interruptions during transitions out of institutional settings [60], and even brief gaps in treatment are associated with elevated risks of overdose and other adverse outcomes [4, 61]. Moreover, participants’ lack of knowledge about how their treatment will be maintained upon leaving also suggests a lack of policies and practices for integration and communication between the residential facility, the methadone providers, and patients.

To mitigate these risks, residential programs could incorporate structured continuation-of-care strategies, including formal linkage-to-care protocols that emphasize direct provider-to-provider communication [62] and are clearly communicated to patients early on in their engagement with the MMU. This is particularly the case since individuals who are transitioning out of residential treatment and back into the community are often overwhelmed with the need to establish housing and employment, attend to family relationships, and make decisions about their future relationship to drug use. For example, scheduling a patient’s first post-discharge dose or outpatient appointment prior to discharge could reduce the likelihood of treatment disruption during this high-risk period. In addition to increasing linkages between the residential facility and community OTP/MMU, treatment support efforts such as providing transportation and logistical assistance [63], and expanded use of peer support to facilitate engagement [64], could all help to ensure that patients maintain MT upon leaving residential care. Without such flexibility, transitions from MMU-based residential care to traditional OTP settings may inadvertently recreate the very barriers that undermine long-term treatment retention. At the same time, it is worth noting that institutional reforms within brick-and-mortar OTPs—particularly the provision of sufficient take-home doses to reduce daily or near-daily commuting requirements—would directly address many of the concerns participants described.

Taken together, the themes identified in this study suggest that mobile methadone units improve patient experiences primarily through three mechanisms: reducing logistical barriers to medication access, minimizing exposure to drug-related triggers commonly encountered near brick-and-mortar clinics, and enabling more personalized interactions with providers. At the same time, the transition from MMU-based care back to traditional OTPs may reintroduce many of the barriers that patients had temporarily avoided. More broadly, these findings underscore the importance of examining how institutional structures shape patients’ everyday experiences of methadone treatment. While regulatory reforms have increasingly focused on expanding access to medication, our results suggest that the settings in which treatment is delivered—and the logistical and social conditions surrounding that delivery—may play an equally important role in shaping engagement and retention.

### Limitations

These findings should be understood in the context of several factors that limit their generalizability. First, since our qualitative data was collected from a convenience sample, our findings are not representative and cannot be generalized to the larger population of people using mobile MT. The logistics of operating in partnership with a residential facility are likely to be different than other settings of care. An MMU serving a residential facility needs only park directly outside to serve its entire patient population and medications are stored and managed by residential treatment staff. But outside of institutional settings, where people are distributed over wide areas, patients would still have to commute to the MMUs location and may not be provided with take-home doses, likely reducing its value to many. Moreover, the convenience sampling strategy we utilized, whereby providers served as recruiters, can introduce selection bias [65, 66] as providers may be more likely to recruit patients with more positive treatment experiences. As such, our data may not include all perspectives, and our sample may be skewed toward patients with more positive relationships with providers and more supportive views of the program. We were also unable to distinguish whether participants initiated MT in the residential treatment center or if they initiated treatment at a brick-and-mortar OTP which would have added greater insight into whether participants used the MMU to replace prior transport-based dosing. Lastly, we were unable to collect participants demographic data; this would have helped to contextualize participant responses and could have suggested avenues for continuing research. These limitations may limit transferability of data to other contexts and should be taken into account when reviewing our findings as well as demonstrating the need for continued empirical research on MMU patient population in the U.S.

## Conclusion

Our findings indicate strong support among MT patients for the MMU model in residential SUD settings and highlight its potential for amending gaps in treatment in other institutional contexts. While previous studies have evaluated the feasibility of mobile methadone programs from a service delivery perspective, this study contributes qualitative evidence from patients themselves regarding how mobile delivery models reshape their experience of treatment. Our findings further underscore the importance of ensuring effective transitions of care from residential treatment to community-based programs that can continue to meet patients’ needs. Taken together, these results contribute to the growing literature on patients’ perspectives of MT and emphasize the value of delivering care in ways that are patient-centered, accessible, and responsive to inequities in access, initiation, and retention.

## Data Availability

The datasets are not publicly available because we did not obtain participant consent for data sharing. They are stored on a protected server.

## Abbreviations

OUD: Opioid use disorder
MMU: Mobile medication units
MT: Methadone treatment
SUD: Substance use disorder
MOUD: Medication for opioid use disorder
OTP: Opioid Treatment Program
